# Transmission of Helminths between Species of Ruminants in Austria Appears More Likely to Occur than Generally Assumed

**DOI:** 10.3389/fvets.2018.00030

**Published:** 2018-03-08

**Authors:** Jakob Winter, Steffen Rehbein, Anja Joachim

**Affiliations:** ^1^Department of Pathobiology, Institute of Parasitology, University of Veterinary Medicine Vienna, Vienna, Austria; ^2^Kathrinenhof Research Center, Boehringer Ingelheim Vetmedica GmbH, Rohrdorf, Germany

**Keywords:** cervidae, bovidae, trematodes, cestodes, nematodes, liability index, biodiversity index, ABOL

## Abstract

Helminth infections of the gastrointestinal tract and lungs can lead to devastating economical losses to the pastoral based animal production. Farm animals can suffer from malnutrition, tissue damage, and blood loss resulting in impaired production traits and reproduction parameters. In Austria, pastures grazed by sheep, goats, and cattle overlap with the habitats of several species of wild cervids (roe deer, red deer, sika deer, and fallow deer) and bovids (mouflon, chamois, and ibex), and transmission of parasites between different ruminant species seems likely. A complete and updated overview on the occurrence of helminths of domestic and wild ruminants in Austria is presented. Based on these data, intersections of the host spectrum of the determined parasites were depicted. The “liability index” was applied to identify the ruminant species, which most likely transmit parasites between each other. A degree for host specificity was calculated for each parasite species based on the average taxonomic distance of their host species. Of the 73 identified helminth species 42 were identified as generalists, and 14 transmission experiments supported the assumed broad host specificity for 14 generalists and 1 specialist helminth species. Overall, 61 helminths were found to infect more than one host species, and 4 were found in all 10 ruminant species investigated. From these analyses, it can be concluded that a number of helminth parasites of the gastrointestinal tract and the lungs are potentially transmitted between domestic and wild ruminants in Austria. For some parasites and host species, experimental evidence is in support for possible transmission, while for other such studies are lacking. Host preference of different genotypes of the same parasite species may have a confounding effect on the evaluation of cross-transmission, but so far this has not been evaluated systematically in helminths in Austria. Further studies focusing on experimental cross-transmission and genetic characterization are needed to define the potential consequences for the epidemiology of those parasites, animal welfare, and economic impact.

## Introduction

Transmission of helminths between different host species is a biological feature that is a key element for the understanding of parasite epidemiology and can play a major role for the design of strategies for the control of parasites of veterinary as well as medical importance. Transmission requires a common environment, where host species share the same resources like pasture or watering holes. In some cases, further requirements are necessary, e.g., the presence of a suitable intermediate host. In this study, we analyzed the occurrence of helminths parasitizing ruminants in Austria and evaluated the extent of parasite cross-transmission between different species of hosts. In Austria, 634.071 ha of land are used as grassland. This includes, as a specific characteristic of the alpine country Austria, 330.545 ha of mountain pasture (excluding unused areas like forests and abandoned land). Almost 2 million cattle, 380,000 sheep and 83,000 goats are reared in Austria, with around 250,000 cattle, 113,000 sheep, and 10,000 goats kept on mountain pasture.^1^ Seven species of wild ruminants form free-living populations in Austria, which include four species of cervids: red deer, roe deer, sika deer, and fallow deer with annual harvests (2015/2016) of 52,024, 276,222, 1,053, and 805 individuals, respectively; and three species of bovids: chamois, mouflon, and ibex with annual (2015/2016) harvests of 20,371, 2,450 and 549 individuals, respectively.^2^

As the habitats of red deer (1), roe deer (2), chamois (3), mouflon (4), and ibex (5) overlap with pasture of livestock (3) and protective fences are rare, contact of a particular ruminant species with the local helminth populations of the abovementioned other species on mountain pasture is possible. Fallow deer prefer habitats below 800 m above sea level (6). Sika deer often occurs in the same habitats as roe deer and fallow deer (7). As the habitat of ibex ranges on average between 1,600 and 3,200 m above sea level (5), contacts between the helminth population of fallow deer and sika deer and the helminth population of ibex are highly unlikely.

To evaluate theoretical transmission scenarios for Austria, we developed a model of likeliness of transmission of helminths between different species of ruminants and compared these findings with the results from transmission experiments described in the international literature.

## Materials and Methods

### Species of Ruminants Included in the Analyses

Our study included cattle (*Bos primigenius taurus*), sheep (*Ovis gmelini aries*), and goat (*Capra aegagrus hircus*) as domestic species and red deer (*Cervus elaphus*), roe deer (*Capreolus capreolus*), sika deer (*Cervus nippon*), fallow deer (*Dama dama*), mouflon (*Ovis gmelini musimon*), ibex (*Capra ibex*), and chamois (*Rupicapra rupicapra*) as wild species.

Red deer, sika deer, fallow deer, and roe deer belong to the ungulate family Cervidae (8). Red deer, sika deer, and fallow deer belong to the tribe Cervini or Old World deer (Plesiometacarpalia) (9, 10). Red deer and sika deer belong to the same genus *Cervus*; they can interbreed and produce fertile offspring (7, 11). Red deer is widespread in Austria from the alpine areas of the west to the eastern Danube flood lands. Sika deer and fallow deer are not native to Austria. Sika deer was introduced to Europe in the mid-nineteenth century. Its distribution in Austria is largely restricted to the lowlands of Lower Austria (12).^2^ Fallow deer was present in Europe until the last ice age and has been reintroduced in the sixteenth to seventeenth century; it also prefers lowland areas (13, 14). Roe deer is the most numerous and widespread wild ruminant species in Austria.^2^ It belongs to the tribe Capreolini or New World deer (Telemetacarpalia) (8).

**Figure 1:**
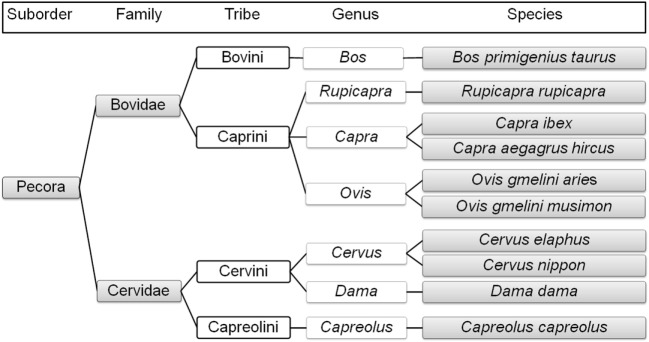
Phylogenetic relationships of the selected ruminant species in Austria (1–8, 11, 14, 15) created with TimeTree; www.timetree.org.

Sheep, mouflon, ibex, goat, chamois, and domestic cattle belong to the family Bovidae. Free-living mouflon, ibex, and chamois are essentially restricted to the higher-altitude regions of Austria. The tribe Caprini includes sheep, mouflon, ibex, goat, and chamois. Sheep and mouflon both belong to the genus *Ovis* and are close relatives, as are ibex and goat (both belonging to the genus *Capra*), while chamois belong to the genus *Rupicapra* (8). Domestic cattle belong to the genus *Bos* within the tribe Bovini (8, 15) (Figure 1).

### Literature Search for Helminths of the Gastrointestinal Tract and Lungs of Ruminants in Austria

Helminth species recorded for the gastrointestinal tract and lungs of ruminants in Austria were summarized from published post mortem records, prevalence surveys (only including those with records of worms determined to species) and experimental transmission studies (Table S1 in Supplementary Material). Reclassification of parasites (synonymization of names and acceptance of the concept of polymorphism) was taken into account. Literature research at the Veterinary Library of the Veterinary University Vienna and using the search engines “PubMed,”^3^ “Scopus,”^4^ and “VetmedSeeker”^5^ provided 27 publications, which ranged from 1969 (16) to 2014 (17).

The animals included in these records were derived from different parts of Austria, both the (pre-)alpine west and the central and eastern lowlands.

### Host Specificity of the Listed Helminths

Inspired by Poulin and Mouillot (18), the biodiversity index (BI) $\bar{\omega }$ (19) was calculated in its general form and used as a measure for host specificity of each parasite (Eq. 1). Its value increases with greater taxonomic distance (genus, tribe, family, and infraorder) which the host species, infected by the parasite in question, have to a common ancestor host and is inversely proportional to host specificity. The BI for two host species which share a common ancestor in the same genus will be 1; it will be 2 for species in the same tribe, 3 for those from the same family, and 4 for those in the same infraorder. Its value will be 0 if there is only one infected host,

(1) $$\Delta^{+}=\bar{\omega }=\frac{\sum \sum_{i\neq j}\omega_{ij}}{s(s-1)}.$$

BI—this formula (19) factors the number of host species used by a parasite (*s*), the average taxonomic distance (ω*_ij_*), which is the mean number of steps up the Linnaean hierarchy (genus, tribe, family, and infraorder) between the host species *i* and *j* {*i*: 1, … *s*; *j*: 1, …, *s, i* ≠ *j*}, that must be taken to reach a taxon, which is common to both host species.

Taxonomic heterogeneity of the parasitized host species is reflected by the standard variance of the BI Λ^+^ (19) (Eq. 2). Its value increases proportionally to the variability in taxonomic distinctness of all infected host species. The variance is by nature not assessable if there is only one infected host species and is 0 if there are only two infected host species or under rare particular taxonomic distance combinations of infected host species.

A generalist parasite was defined to have the ability to infect host species which have common ancestors in the same host family or infraorder. A minimum of three infected host species was set to increase the informative value. Thus, the generalist parasite BI is >2 and its standard variance >0. Specific parasites infect host species with a common ancestor within the same tribe. Their BI value is ≤2, and its standard variance >0,

(2) $$\Lambda^{+}=\frac{\sum \sum_{i\neq j}{\left(\omega_{ij}-\bar{\omega }\right)}^{2}}{s(s-1)}.$$

Standard variance of the BI: *s*: number of host species that are infected by the parasite in question, *i* and *j*: taxonomic distance between host species {*i*: 1, … 4; *j*: 1, …, 4, *i* ≠ *j*}, ω*_ij_*: average taxonomic distance, $\bar{\omega }$: average taxonomic distinctness (≜BI).

### Susceptibility of Host Species to Be Infected with Generalist Parasites of Other Hosts

The liability index *L* (20) was applied to measure the degree to which each ruminant species in Austria is susceptible to infection with generalist helminths from another ruminant species (Eq. 3). The value of the *L* index ranges from −1 to 1. Hosts with an entirely unique parasite spectrum have a liability index of −1, hosts with half-unique and half-shared parasites with another host have an index of 0, and those hosts which share all their parasites with another host have an index of 1 (20). The liability index was calculated for all ruminant species in Austria. Calculations were done with RStudio (21),

(3) $$L=\frac{S_{AB-}U_{A}}{d_{A}}.$$

Liability index (20)—this formula calculates the index by dividing the difference of the number of shared parasites (*S_AB_*) by one selected host species (*A*) with another host species (*B*), and the number of parasites unique to that selected host species (*U_A_*) by the total number of parasites of that selected host species (*d_A_*).

### Likelihood of Parasites to Occur in Selected Ruminant Species

Liability index, BI, and standard variance of the BI were combined to assess which ruminants are likely to be hosts for the same helminths.

It was assumed that generalist parasites of a selected host species will occur in other ruminant species if their liability index is >0. Specific parasites of a selected host species are expected to occur in other ruminants if the liability index is >0 and the host species are members of the same tribe/genus. An adapted liability index L* (Eq. 4) was developed which takes this assumed situation into account. Numbers of shared generalist, shared specialist, unique generalist and unique specialist (US) parasites were calculated between pairs of ruminant species, sharing a liability index ≥0. Parasites which could not be clearly attributed to generalist and specialist parasites were excluded from the calculation,

(4) $$L*=\frac{{\text{SG}}_{AB}+{\text{SS}}_{AB}+{\text{UG}}_{A}-{\text{US}}_{A}}{{\text{SG}}_{AB}+{\text{SS}}_{AB}+{\text{UG}}_{A}+{\text{US}}_{A}}.$$

Adapted liability index for ruminants of different tribes with a liability index ≥0, *A, B*: compared ruminant species, SG*_AB_*: shared generalist parasites, SS*_AB_*: shared specialist parasites, UG*_A_*: unique generalist parasites, and US*_A_*: unique specialist parasites.

As US parasites are seen to be occurring in the same tribe/genus, the adapted liability index will be 1 for a pair of ruminant species of the same tribe/genus.

Furthermore, an adapted ratio for shared generalist parasites (RSG*_AB_*) and shared specialist parasites (RSS*_AB_*) was calculated to determine which of the two groups of helminths are mainly shared (Eq. 5). The results give additional indication which parasites are likely to be shared between two ruminant species,

(5) $${\text{RSG}}_{AB}=\frac{{\text{SG}}_{AB}}{{\text{SG}}_{AB}+{\text{SS}}_{AB}+{\text{UG}}_{A}};{\text{RSS}}_{AB}=\frac{{\text{SS}}_{AB}}{{\text{SG}}_{AB}+{\text{SS}}_{AB}+{\text{UG}}_{A}}.$$

Ratio of shared generalist (RSG*_AB_*) and ratio of shared specialist (RSS*_AB_*): *A, B*: compared ruminant species, SG*_AB_*: shared generalist parasites, SS*_AB_*: shared specialist parasites, and UG*_A_*: unique generalist parasites.

## Results

### Helminth Parasites of the Gastrointestinal Tract and Lungs of Ruminants in Austria

**Figure 2:**
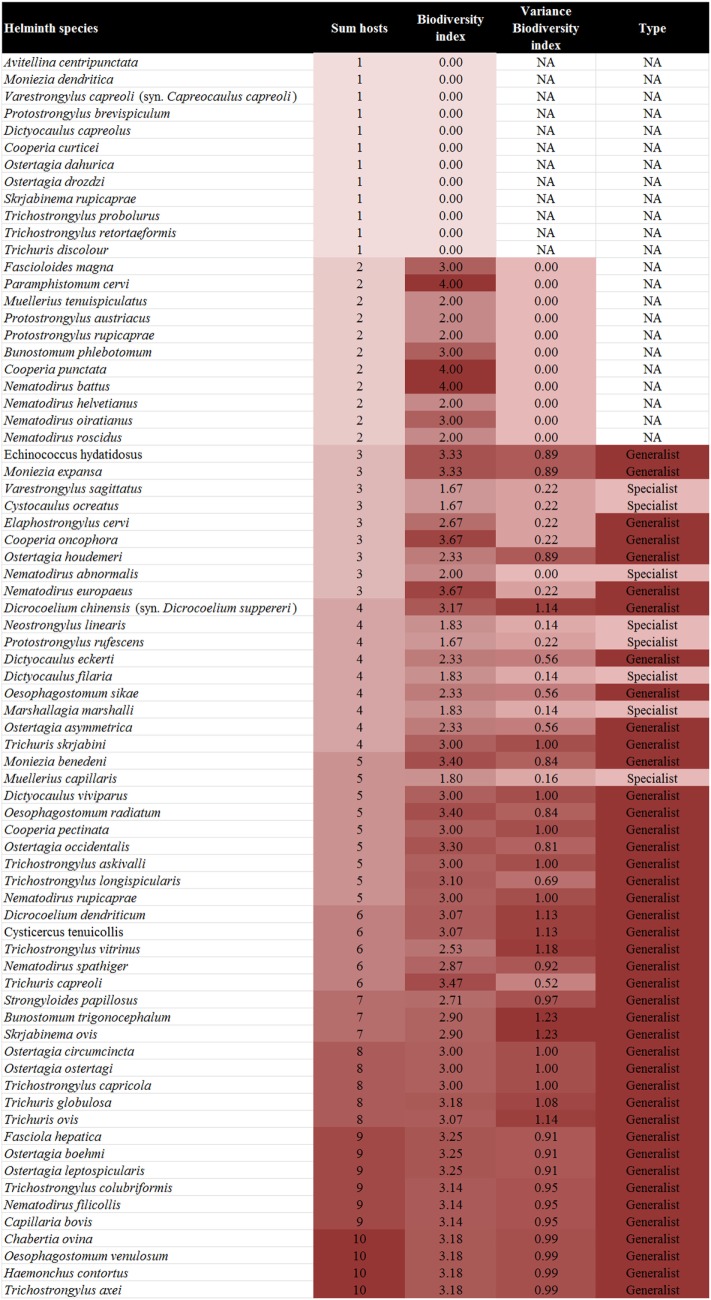
Biodiversity index, variance of the biodiversity index, and type of the different helminth species by ascending number of host species.

In total, 73 helminth species of the gastrointestinal tract and lung of ruminants in Austria were recorded from the literature, belonging to the families Fasciolidae (2 species), Dicrocoeliidae (2), Paramphistomidae (1), Taeniidae (2; only larval stages), Anoplocephalidae (4), Strongyloididae (1), Protostrongylidae (11), Dictyocaulidae (4), Chabertiidae (4), Ancylostomatidae (2), Trichostrongylidae (25), Molineidae (9), and Trichuridae (6) (Figure 2).

### Host Range of the Helminth Parasites Recorded

Of the 73 helminths species recorded in 10 ruminant species in Austria 21 (28.8%; sika deer) to 45 (61.6%; roe deer) have been described per ruminant host. The individual parasite species were recorded in 1–10 host species (Figure 2).

### Host Specificity of the Helminth Parasites Recorded

The BI of the helminths ranged from 0 (*n* = 12) for species with only one host listed to four (*n* = 3) for helminths occurring in two hosts belonging to different families. The standard variance of the BI ranged from 0 (*n* = 12), for helminths infecting only two hosts (and in one case three hosts, *Nematodirus abnormalis*, due to the combination of hosts) to 1.23 for helminths (*Bunostomum trigonocephalum* and *Skrjabinema ovis*) infecting a range of distantly related hosts (Figure 2).

In total, 42 of 73 helminths (54.5%) had a BI > 2 with a variance of >0 and were therefore defined as generalist parasites, while seven helminth species (9.1%) had a BI ≤ 2 with a variance of >0 and therefore were defined as specialist parasites. Out of these, six species occur in the tribe Caprini and one in the tribe Cervini. One helminth species, *N. abnormalis*, had a BI ≤ 2 with a variance of 0 but was described from three different hosts and was therefore regarded as a specialist parasite. No clear attribution was possible for 23 (31.5%) species (Figure 2).

For a number of helminths cross-infection between ungulate species was confirmed in experimental studies; this concerned 17 generalist species, 1 specialist (*Muellerius capillaris*), and 4 species without clear host-type specificity (Table 1).

**Table 1:** Parasites that have been successfully transmitted between ruminants in comparison with the calculated biodiversity index (BI), variance of the biodiversity index (VBI), and host specificity type (type—G, generalist; S, specialist; NA, not applicable).

Helminth species	Cattle	Sheep	Mouflon	Ibex	Goat	Chamois	Roe deer	Red deer	Fallow deer	Sika deer	BI	VBI	Type
**Cestoda**													
Anoplocephalidae													
*Moniezia benedeni*	9	9									3.40	0.84	G

**Nematoda**													
Strongyloididae													
*Strongyloides papillosus*	3, 8	3, 8, 13	13		13						2.71	0.97	G

**Protostrongylidae**													
*Muellerius capillaris*		4			4						1.80	0.16	S

**Dictyocaulidae**													
*Dictyocaulus eckerti*	2								2		2.33	0.56	G
*Dictyocaulus viviparus*	2, 5, 7						7	5, 7	2, 5		3.00	1.00	G
Chabertiidae													
*Oesophagostomum venulosum*	3, 7, 12	1, 3						1, 7, 12			3.18	0.99	G

**Trichostrongylidae**													
*Cooperia curticei*	3, 6, 8, 12	3, 6, 8, 11						11, 12			0.00	NA	NA
*Cooperia oncophora*	3, 6, 9, 12	3, 6, 9					1	12			3.67	0.22	G
*Cooperia pectinata*	8	8									3.00	1.00	G
*Cooperia punctata*	3, 8, 12	3, 8						12			4.00	0.00	NA
*Haemonchus contortus*	3, 8, 10, 12	1, 3, 8, 10, 11, 13	7, 13		11, 13		1, 7, 13	7, 11, 12	7		3.18	0.99	G
*Ostertagia leptospicularis*	1, 7	1, 13			1, 13		1, 7, 13	7			3.25	0.91	G
*Ostertagia boehmi*	7	1			13		1, 7, 13	7			3.25	0.91	G
*Ostertagia circumcincta*	3	3, 11, 13	13		13			11			3.00	1.00	G
*Ostertagia ostertagi*	3, 12	3						12			3.00	1.00	G
*Trichostrongylus axei*	8, 10, 12, 13	8, 10, 13	13		13		13	12			3.18	0.99	G
*Trichostrongylus colubriformis*	1, 12	1, 13	13		1, 13		1, 13	12			3.14	0.95	G
*Trichostrongylus vitrinus*	3	1, 3			1, 13		13				2.53	1.18	G

**Molineidae**													
*Nematodirus helvetianus*	8, 10	8, 10									2.00	0.00	NA
*Nematodirus roscidus*	7							7			2.00	0.00	NA
*Nematodirus rupicaprae*											3.00	1.00	G

**Trichuridae**													
*Trichuris capreoli*	7							7			3.47	0.52	G
*Trichuris ovis*	8, 9	8, 9									3.07	1.14	G

**Number** 1 2 3 4 5 6 7	**Citation** Barth and Dollinger (22) Bienioschek et al. (24) Borgsteede (26) Ten Doesschate et al. (28) Foreyt et al. (30) Johnson et al. (32) Koutz and Rebrassier (34)			**Number** 8 9 10 11 12 13					**Citation** Kutzer (23) Porter (25) Smith and Archibald (27) Stoll (29) Tapia-Escárate et al. (31) Wetzel and Fortmeyer (33)				

### Susceptibility of Host Species to Helminth Parasites

The liability index for the one-to-one comparison between individual ruminant species ranged from −0.55 (goat–sika deer) to 1.000 (goat–sheep and fallow deer–roe deer) with a mean of 0.19 and a median of 0.13. The mean number of helminth species shared between two ruminant species was 19 (range 5–34, median: 19), the mean number of unique helminth species was 14 (range 0–33, median: 13) (Table 2).

**Table 2:** Shared and unique helminths species and liability indices for the pairs of ruminant species included in the present study.

	Shared	Shared G	Shared S	S, no clear attribution	Unique	Unique G	Unique S	Unique, no clear attribution	D	Ratio shared G:S	Ratio shared S:G	*L*	L*
Cattle–sheep	19	18	0	1	7	4	0	3	26	1	0	0.46	1
Cattle–mouflon	16	16	0	0	10	6	0	4	26	1	0	0.23	1
Cattle–ibex	14	14	0	0	12	8	0	4	26	1	0	0.08	1
Cattle–goat	13	13	0	0	13	9	0	4	26	1	0	0.00	1
Cattle–chamois	19	18	0	1	7	4	0	3	26	1	0	0.46	1
Cattle–roe deer	22	21	0	1	4	1	0	3	26	1	0	0.69	1
Cattle–fallow deer	13	13	0	0	13	9	0	4	26	1	0	0.00	1
Cattle–red deer	19	19	0	0	7	3	0	4	26	1	0	0.46	1
Cattle–sika deer	11	11	0	0	15	11	0	4	26	1	0	−0.15	NA
Sheep–cattle	19	18	0	1	21	10	6	5	40	1	0	−0.05	NA
Sheep–mouflon	27	22	5	0	13	6	1	6	40	0.85	0.15	0.35	1
Sheep–ibex	28	23	4	1	12	5	2	5	40	0.88	0.13	0.40	1
Sheep–goat	22	17	5	0	18	11	1	6	40	0.85	0.15	0.10	1
Sheep–chamois	31	26	4	1	9	2	2	5	40	0.88	0.13	0.55	1
Sheep–roe deer	26	26	0	0	14	2	6	6	40	1	0	0.30	0.647
Sheep–fallow deer	13	12	0	1	27	16	6	5	40	1	0	−0.35	NA
Sheep–red deer	22	22	0	0	18	6	6	6	40	1	0	0.10	0.647
Sheep–sika deer	10	10	0	0	30	18	6	6	40	1	0	−0.50	NA
Mouflon–cattle	16	16	0	0	16	10	5	2	32	1	0	0.00	NA
Mouflon–sheep	27	22	5	0	5	4	0	2	32	0.84	0.16	0.69	1
Mouflon–ibex	23	20	3	0	9	6	1	3	32	0.9	0.1	0.44	1
Mouflon–goat	21	17	4	0	11	9	1	2	32	0.87	0.13	0.31	1
Mouflon–chamois	27	24	3	0	5	2	2	2	32	0.9	0.1	0.69	1
Mouflon–roe deer	26	26	0	1	6	0	5	1	32	1	0	0.63	0.677
Mouflon–fallow deer	14	14	0	0	18	12	5	2	32	1	0	−0.13	NA
Mouflon–red deer	22	21	0	1	10	5	5	1	32	1	0	0.38	0.677
Mouflon–sika deer	11	11	0	0	21	15	5	2	32	1	0	−0.31	NA
Ibex–cattle	14	14	0	0	19	9	4	6	33	1	0	−0.15	NA
Ibex–sheep	28	23	4	1	5	0	0	5	33	0.85	0.15	0.70	1
Ibex–mouflon	23	20	3	0	10	3	1	6	33	0.88	0.12	0.39	1
Ibex–goat	18	15	3	0	15	8	1	6	33	0.88	0.12	0.09	1
Ibex–chamois	32	23	3	6	1	0	1	0	33	0.88	0.12	0.94	1
Ibex–roe deer	23	23	0	0	10	0	4	6	33	1	0	0.39	0.704
Ibex–fallow deer	11	11	0	0	22	12	4	6	33	1	0	−0.33	NA
Ibex–red deer	18	18	0	0	15	5	4	6	33	1	0	0.09	0.704
Ibex–sika deer	8	8	0	0	25	15	4	6	33	1	0	−0.52	NA
Goat–cattle	13	13	0	0	9	4	5	0	22	1	0	0.18	0.545
Goat–sheep	22	17	5	0	0	0	0	0	22	0.77	0.23	1.00	1
Goat–mouflon	21	17	4	0	1	0	1	0	22	0.81	0.19	0.91	1
Goat–ibex	18	15	3	0	4	2	2	0	22	0.85	0.15	0.64	1
Goat–chamois	20	17	1	2	2	0	2	0	22	0.94	0.06	0.82	1
Goat–roe deer	17	17	0	0	5	0	5	0	22	1	0	0.55	0.545
Goat–fallow deer	9	9	0	0	13	8	5	0	22	1	0	−0.18	NA
Goat–red deer	14	14	0	0	8	3	5	0	22	1	0	0.27	0.545
Goat–sika deer	5	5	0	0	17	12	5	0	22	1	0	−0.55	NA
Chamois–cattle	19	18	0	1	25	13	4	8	44	1	0	−0.14	NA
Chamois–sheep	31	26	4	1	13	5	0	8	44	0.89	0.11	0.41	1
Chamois–mouflon	27	24	3	0	17	7	1	9	44	0.91	0.09	0.23	1
Chamois–ibex	32	23	3	6	12	8	1	3	44	0.91	0.09	0.45	1
Chamois–goat	20	17	3	0	24	14	1	9	44	0.91	0.09	−0.09	NA
Chamois–roe deer	30	30	0	0	14	1	4	9	44	1	0	0.36	0.771
Chamois–fallow deer	14	14	0	0	30	17	4	9	44	1	0	−0.36	NA
Chamois–red deer	24	24	0	0	20	7	4	9	44	1	0	0.09	0.771
Chamois–sika deer	11	11	0	0	33	20	4	9	44	1	0	−0.50	NA
Roe deer–cattle	22	21	0	1	23	15	4	6	45	1	0	−0.02	NA
Roe deer–sheep	26	26	0	0	19	10	4	7	45	1	0	0.16	0.8
Roe deer–mouflon	26	26	0	1	19	10	4	6	45	1	0	0.16	0.8
Roe deer–ibex	23	23	0	0	22	13	4	7	45	1	0	0.02	NA
Roe deer–goat	17	17	0	0	28	19	4	7	45	1	0	−0.24	NA
Roe deer–chamois	30	30	0	0	15	6	4	7	45	1	0	0.33	0.8
Roe deer–fallow deer	21	18	3	0	24	18	0	8	45	0.92	0.08	−0.07	NA
Roe deer–red deer	34	29	4	1	11	7	1	5	45	0.9	0.1	0.51	0.951
Roe deer–sika deer	20	16	4	0	25	20	0	7	45	0.9	0.1	−0.11	NA
Fallow deer–cattle	13	13	0	0	12	5	5	4	25	1	0	0.04	NA
Fallow deer–sheep	13	12	0	1	12	6	5	3	25	1	0	0.04	NA
Fallow deer–mouflon	14	14	0	0	11	4	5	4	25	1	0	0.12	0.565
Fallow deer–ibex	11	11	0	0	14	7	5	4	25	1	0	−0.12	NA
Fallow deer–goat	9	9	0	0	16	9	5	4	25	1	0	−0.28	NA
Fallow deer–chamois	14	14	0	0	11	4	5	4	25	1	0	0.12	0.565
Fallow deer–roe deer	25	18	3	6	0	0	1	−1	25	0.86	0.14	1.00	0.909
Fallow deer–red deer	22	18	4	0	3	0	1	4	25	0.82	0.18	0.76	1
Fallow deer–sika deer	16	12	4	0	9	6	0	5	25	0.82	0.18	0.28	1
Red deer–cattle	19	19	0	0	20	12	4	4	39	1	0	−0.03	NA
Red deer–sheep	22	22	0	0	17	9	4	4	39	1	0	0.13	0.771
Red deer–mouflon	22	21	0	1	17	10	4	3	39	1	0	0.13	0.771
Red deer–ibex	18	18	0	0	21	13	4	4	39	1	0	−0.08	NA
Red deer–goat	14	14	0	0	25	17	4	4	39	1	0	−0.28	NA
Red deer–chamois	24	24	0	0	15	7	4	4	39	1	0	0.23	0.771
Red deer–roe deer	34	29	3	2	5	2	1	2	39	0.91	0.09	0.74	0.943
Red deer–fallow deer	22	17	5	0	17	13	0	4	39	0.86	0.14	0.13	1
Red deer–sika deer	20	15	4	1	19	16	0	3	39	0.89	0.11	0.03	1
Sika deer–cattle	11	11	0	0	10	5	5	0	21	1	0	0.05	0.524
Sika deer–sheep	10	10	0	0	11	6	5	0	21	1	0	−0.05	NA
Sika deer–mouflon	11	11	0	0	10	5	5	0	21	1	0	0.05	0.524
Sika deer–ibex	8	8	0	0	13	8	5	0	21	1	0	−0.24	NA
Sika deer–goat	5	5	0	0	16	11	5	0	21	1	0	−0.52	NA
Sika deer–chamois	11	11	0	0	10	5	5	0	21	1	0	0.05	0.524
Sika deer–roe deer	20	16	4	0	1	0	1	0	21	0.8	0.2	0.90	1
Sika deer–fallow deer	16	12	3	1	5	4	1	0	21	0.84	0.16	0.52	1
Sika deer–red deer	20	15	5	0	1	1	0	0	21	0.76	0.24	0.90	1

G, generalist; S, specialist; L, liability index; L*, adapted liability index.

For 60 out of 90 compared ruminant species pairs the liability index was ≥0. The mean number of shared generalist helminth species was 17 (range 5–30, median: 17), the mean number of shared specialists was 1.17 (range 0–5, median: 0), the mean number of unique generalists was 8.4 (range 0–23, median: 8.0), and the mean number of USs was 1.8 (range 0–6, median: 1). The adapted liability index (L*) for the 59 ruminant combinations ranged from 0.55 (goat–cattle, goat–roe deer, and goat–red deer) to 1.0 (*n* = 23 pairs of ruminants) with a mean of 0.9 and a median of 0.94. No calculation of an adapted liability index was possible for 27 species pairs (Table 2).

The ratio of shared generalist helminth species between ruminant species pairs ranged from 0.77 (goat–sheep) to 1.0 (*n* = 58.0 species pairs) with a mean of 0.96 and median of 1. The ratio of shared specialist helminths between ruminant species pairs ranged from 0 (*n* = 58 pairs) to 0.23 (goat–sheep) with a mean of 0.04 and median of 0 (Table 2).

## Discussion

The transmission of pathogens including helminths between host species of different taxa has implications in several directions, including ecology and epidemiology. In our work, we addressed the question whether possible transmission of helminths from one host species to another sharing the same environment can be estimated by calculating different indices, testing this assumption by comparison with the available literature.

Liability index (20), BI, and standard variance of the BI (19) were calculated to determine which helminth species are likely to occur in the most abundant ruminant species in Austria.

Literature search focused only on reports of helminths in these species in Austria and provided 27 articles dating back to the year 1969. As actual comprehensive data about helminth parasites of ruminants in Austria is scarce, we first aimed to provide an overview on this. The results of our analysis suggest, however, that more helminth species may occur and that the host range of described helminth species is larger than described so far. Since the number of available publications for Austria was rather low the evaluation of the data must be seen as preliminary; nevertheless we could provide calculations which can be used to test hypotheses arising from them.

The liability index (20) was used to test which pairs of hosts are most likely to be susceptible for the same parasites. In general, the results of the liability index calculation were as expected: phylogenetically closely related ruminants with similar habitat requirements (e.g., sheep and mouflon) share a high liability index value (≥0.5 = a ruminant pair shares ≥ 75% of their parasites), whereas phylogenetically more distant ruminants with distinct habitats (e.g., chamois and sika deer) share a low liability index (≤0 = a ruminant pair shares less than 50% of all parasites). The outcome of this analysis was unexpectedly low for the pairs cattle–goat (0) and sheep–cattle (−0.05). As the liability index is influenced by the number of helminth species detected which is correlated with the number of hosts examined (e.g., fallow deer and mouflon are less well examined than other species), an adaptation of the liability index was developed. It was hypothesized that ruminant species pairs with a liability index ≥0 will additionally share generalist parasites which have the ability to infect hosts belonging to different host families. Furthermore, it was assumed that ruminant species pairs belonging to the same tribe/genus with a liability index ≥0 will additionally share specific helminths which have the ability to infect closely related host species.

As a measure of host specificity, the BI and its standard variance (18, 19) were calculated for the 73 described helminth species. It can essentially be assumed that host specificity of parasites increases with decreasing taxonomic distinctness between their host species (18). Out of 73 helminth species recorded, 42 were identified as generalists, and 8 were classified as specialists. Four of the generalist species (*Chabertia ovina, Oesophagostomum venulosum, Haemonchus contortus*, and *Trichostrongylus axei*) were described in all 10 ruminants occurring in Austria. Overall, 38 parasites may have the ability to infect more ruminant species than previously described. These should be in the focus for further investigations.

The adapted liability index was calculated for 59 pairs of ruminant species (liability index ≥0), by including the 42 generalist helminths and 8 specialist helminth species. Compared with the (conventional) liability index, the results of the adapted liability index provided higher values in all categories. An increase compared with the value of the conventional liability index was expected, as mainly generalist helminth species were identified in ruminants in Austria. From these findings we conclude that Austrian ruminants have a far more higher risk to be infected by generalist helminths than previously reported [see Table S1 in Supplementary Material for literature and Ref. (16)].A particularly interesting result of this analysis is that based on these calculations cattle were shown to be susceptible to all generalist parasites of all other ruminants in Austria.

The ratio of shared generalists and specialists also revealed tendencies of specialist helminths to occur in a pair of ruminant species. Generally, the ratio shows that mainly generalists (range: 0.77–1.00; mean: 0.96), and only a few specialists (range: 0.00–0.23; mean: 0.04) are shared by ruminant species pairs in Austria. The latter finding indicates the difficulties in unequivocal assigning “specialism” or “generalism” to a particular helminth species.

Overview about the helminth fauna of wild and domestic ruminants in Austria is provided in older literature (23, 35, 36). Additional work on analyzing the helminth fauna of ruminants in the country is necessary to complete the helminth inventory of Austrian ruminants and to further test the hypothesis arising from our analyses.

From an epidemiological point of view, (pre-)alpine mountain pastures can be considered as environment where transmission of helminth parasites between the same and different species are fostered by different factors. The practice of pasturing cattle, goats, and sheep originating from different farms and regions on shared mountain pasture over centuries favors the occurrence of a divergent local population of parasitic helminths. Wild ruminant species that occur in these habitats, such as red deer, chamois, and ibex, may further contribute to the cross-transmission of helminths in such areas by increasing the number of helminth species and sustaining infection cycles.

By contrast, lowland pasture areas of central and eastern Austria are mostly grazed by single species and are usually fenced, limiting the contact between domestic and wild ruminants. Differing spatial host distribution patterns limit the availability of hosts in specific locations, and the actual infection status of a host species may therefore be very different from what is proposed by a model only taking into account susceptibility and not spatial distribution of the host(s) (37–39). While some of the wild ungulate species included in the present study have a rather limited range (s. introduction) others are widely distributed in Austria; however, data on the exact range and density of wild ungulate species are not available and therefore could not be included in the index calculations.

From an epidemiological point of view, the specific risk for a herd or flock of domestic ruminants to become infected with helminths derived from other host species is therefore determined by the presence or absence of the latter in a particular area. This is also highlighted by the unexpectedly low *L* index for cattle-goat (Table 2), as these species are usually not pastured together. Thus, management of domestic animals (including range restrictions) can be a confounding factor for the estimation of the actual transmission risk between these hosts. By contrast, wild ruminants are free-ranging and may graze large geographic areas including domestic animals’ pastures where it is frequently observed (40).

As the occurrence of the same parasites in different host species does not necessarily indicate transmission from one species to another (22), the calculated scenarios were compared with the results of experimental infection studies. In summary, results of 14 transmission experiments [cf. Table 1 and Ref. (41), for literature] support the calculated model. Especially, trichostrongylids (most of all *H. contortus*) could frequently be transmitted between different species of domestic and wild ungulates, but also *O. venulosum, Nematodirus helvetianus*, and *Trichuris ovis* and lungworms of the genus *Dictyocaulus* were cross-transmitted. Red deer and roe deer were most frequently implemented in the cross-transmission between wild and domestic ruminants (Table 1). Apart from the host species mentioned in this study, *M. capillaris* was transmissible from goats to bighorn sheep (30) and from white-tailed deer (*Odocoileus virginianus*) to cattle and lambs in an inoculation experiment (41).

In total, 22 helminths (Table 1) were successfully transmitted between several ruminant species. Seventeen out of these 22 helminth species were previously defined as generalists, one species (*M. capillaris*) was transmitted between sheep and goat and previously identified as tribe specialist. *Cooperia curticei* and *Cooperia punctata*, which were unassigned in our prediction due to the lack of data, were attributed further to the generalists, as successful transmission between cattle, sheep, and red deer was shown (25, 26, 28, 34, 42). No attribution could be done for two helminths, *Nematodirus roscidus* and *N. helvetianus*. Overall the results of the transmission experiments are in accordance with the predictions made in the present analysis, but further analyses will be necessary to evaluate and confirm all cases properly.

Since many helminth taxa have notoriously poor morphological characteristics (even at the adult stage) misidentification should be considered as a potential confounding effect of studies determining the helminth fauna of wildlife. DNA barcoding could provide additional data that can be used to exactly determine the helminth fauna of different hosts but data are scarce (43). Cryptic parasite species have been suggested previously [for review, see Ref. (44)], including species of trichostrongylids from ungulates (45–47). Especially specimens of the *Trichostrongylus* and members of the Ostertagiinae subfamily are notoriously difficult to determine to species level, and a number of different names (valid or invalid) can be found in the literature which further complicate species assignment (48). *T. axei* seems to be a real generalist as has been shown by transmission experiments (see above), and this is corroborated by its genetic population structure with high diversity and high gene flow between sympatric hosts (49). In lungworms of the genus *Dictyocaulus* from wild ungulates from Hungary different gene flow rates suggested different host range capacities in the three species *Dictyocaulus eckerti, Dictyocaulus capreolus*, and *Dictyocaulus viviparus* with *D. eckerti* being the most generalist species, while *D. capreolus* seems to have a cryptic genetic structure (50). As red deer was shown to harbor lungworms, which were genetically distinct from the other *Dictyocaulus* species, a new name, *Dictyocaulus cervi*, was proposed recently (51).

Although considerable genetic differences were found in *H. contortus* of sheep, goat, chamois, roe deer, and ibex in alpine areas, the lack of correlation between different mitochondrial clusters with host species indicates that *H. contortus* is a generalist parasite species (52).

Another genus that is known for its diversity despite morphological similarities is *Trichuris*, and it has previously been suggested that *T. discolor* may be a species complex (53).

In the trematode species found in our search, different levels of genetic diversity were found; *Fasciola hepatica* is highly divergent (54) whereas *Fascioloides magna* is an imported fluke in Austria (55) with limited genetic diversity (56).

These examples of differing genetic population structures of helminths, implying the presence of cryptic species with different host preferences, are not exhaustive but highlight the difficulties in determining the exact associations of species/genotypes to host species and the underlying transmission scenarios. Genotyping seems to be the appropriate prerequisite for correct assignment of parasites to their hosts. The “Austrian Barcode of Life” initiative is dedicated to barcode all major taxa of animals, including helminths, in Austria (ABOL^6^). It has contributed so far with 112 sequences from 23 different helminths for DNA barcodes at the cytochrome oxidase I mitochondrial locus (compared with 5 sequences that were available for Austria in the Barcode of Life Data Systems^7^).

With the present study and the proposed model we intend to guide future research interest toward the generalist helminths species of ruminants in Austria to unravel their true host associations and population structures. This is not only of ecological interest but also of relevance for veterinary medicine as the effective control of helminths in livestock also depends on the range of parasites in wildlife. In principle, wild ungulates are not legally accessible for anthelmintic treatment [although exceptions have been made for, e.g., control of *F. magna* in Austria, see Ref. (55)]. Therefore pasture contamination by helminth eggs or larvae from feces of, e.g., free-ranging deer may be considerable and must be taken into account when judging infection risks for domestic livestock. This is particularly important in areas where unfenced pastures are utilized for transhumance in alpine regions, but also applies to lowland pasture with high densities of roe deer populations crossing fields regularly. Such interphases between wild and domestic animals are of particular importance for the exchange of parasite communities and host utilization by parasites and should also be in the focus of studies testing the proposed model for ruminant helminths in Austria.

## Author Contributions

JW, SR, and AJ planned the project; JW drafted the manuscript, AJ and SR revised it. All the authors agreed on the final version of the submitted manuscript.

## Conflict of Interest Statement

The authors declare that the research was conducted in the absence of any commercial or financial relationships that could be construed as a potential conflict of interest.

## Footnotes

^1^www.agraroekonomik.at/index.php?id=gruenerbericht2016&no_cache=1.

^2^www.statistik.at/web_de/statistiken/wirtschaft/land_und_forstwirtschaft/viehbestand_tierische_erzeugung/jagd/index.html.

^3^www.ncbi.nlm.nih.gov/pubmed.

^4^www.scopus.com.

^5^www.vetmeduni.ac.at/bibl/vetmedseeker.

^6^www.abol.ac.at/.

^7^www.boldsystems.org/.
