# Supplementary material for: Transmission of Helminths between Species of Ruminants in Austria Appears More Likely to Occur than Generally Assumed
Source: Front Vet Sci. 2018 Mar 8;5:30. doi: 10.3389/fvets.2018.00030 (PMC5890149; doi:10.3389/fvets.2018.00030)
Supplement: Supplementary file 1 [file table_1.docx]

Supplementary Table 1: Overview of the helminths species and their host species described for Austria (Kutzer et al., 1987, 1988: for Hungary, included due to the close locations of the investigated area to Austria).

| **No.** | **Helminth species** | **Cattle** | **Sheep** | **Mouflon** | **Ibex** | **Goat** | **Chamois** | **Roe deer** | **Red deer** | **Fallow deer** | **Sika deer** |
| --- | --- | --- | --- | --- | --- | --- | --- | --- | --- | --- | --- |
|  | **TREMATODA** |  |  |  |  |  |  |  |  |  |  |
|  | **Fasciolidae** |  |  |  |  |  |  |  |  |  |  |
| 1 | *Fasciola hepatica* | Halabi et al., 1997 | Feichtenschlager et al., 2014; Rehbein and Winter, 1999 | Hille, 2004; Kutzer and Hinaidy, 1969 |  | Otranto et al., 2007 | Kutzer and Hinaidy, 1969; Prosl, 1973 | Kutzer and Hinaidy, 1969 | Kutzer and Hinaidy, 1969 | Kutzer, 1988; Rehbein et al., 2014 | Rehbein, 2010 |
| 2 | *Fascioloides magna* |  |  |  |  |  |  | Sattmann et al., 2014 | Prosl, 1973; Sattmann et al., 2014 |  |  |
|  | **Dicrocoeliidae** |  |  |  |  |  |  |  |  |  |  |
| 3 | *Dicrocoelium chinensis* (syn. *Dicrocoelium suppereri*) |  |  | Hinaidy, 1983 |  |  |  | Hinaidy, 1983 | Prosl, 1973 |  | Otranto et al., 2007; Rehbein, 2010; Rehbein and Visser, 2007 |
| 4 | *Dicrocoelium dendriticum* |  | Feichtenschlager et al., 2014; Rehbein and Winter, 1999 | Hille, 2004; Kutzer and Hinaidy, 1969 | Prosl, 1973 |  | Kutzer and Hinaidy, 1969; Prosl, 1973 | Kutzer and Hinaidy, 1969 | Kutzer and Hinaidy, 1969 |  |  |
|  | **Paramphistomidae** |  |  |  |  |  |  |  |  |  |  |
| 5 | *Paramphistomum cervi* |  |  | Hille, 2004; Kutzer and Hinaidy, 1969 |  |  |  |  | Kutzer and Hinaidy, 1969 |  |  |
|  | **CESTODA** |  |  |  |  |  |  |  |  |  |  |
|  | **Taeniidae (metacestodes)** |  |  |  |  |  |  |  |  |  |  |
| 6 | Cysticercus tenuicollis |  | Kutzer and Hinaidy, 1969 | Hille, 2004; Kutzer and Hinaidy, 1969 | Prosl, 1973 |  | Feldbacher, 1979; Prosl, 1973 | Kutzer and Hinaidy, 1969 |  |  | Kutzer and Hinaidy, 1969 |
| 7 | Echinococcus hydatidosus |  |  | Hille, 2004 |  |  | Feldbacher, 1979; Prosl, 1973 | Schwarz et al., 2011 |  |  |  |
|  | **Anoplocephalidae** |  |  |  |  |  |  |  |  |  |  |
| 8 | *Avitellina centripunctata* |  |  |  |  |  | Feldbacher, 1979; Prosl, 1973 |  |  |  |  |
| 9 | *Moniezia benedeni* | Böhm, 1987; Velarde, 1977 | Rehbein and Winter, 1999 |  |  |  |  | Kutzer and Hinaidy, 1969; Rehbein and Winter, 1999 | Kutzer and Hinaidy, 1969 |  | Rehbein, 2010 |
| 10 | *Moniezia dendritica* |  |  |  |  |  |  |  | Prosl, 1973 |  |  |
| 11 | *Moniezia expansa* |  | Prosl, 2009 (pers. comm.) |  |  |  | Kutzer and Hinaidy, 1969; Prosl, 1973 |  | Kutzer and Hinaidy, 1969 |  |  |
|  | **NEMATODA** |  |  |  |  |  |  |  |  |  |  |
|  | **Strongyloididae** |  |  |  |  |  |  |  |  |  |  |
| 12 | *Strongyloides papillosus* | Kutzer, 1969 | Kutzer, 1988 | Hille, 2004; Kutzer and Hinaidy, 1969 | Kutzer and Hinaidy, 1969; Prosl, 1973 | Kutzer, 1988 | Kutzer, 1988 | Kutzer and Hinaidy, 1969 |  |  |  |
|  | **Protostrongylidae** |  |  |  |  |  |  |  |  |  |  |
| 13 | *Varestrongylus capreoli* (syn. *Capreocaulus capreoli*) |  |  |  |  |  |  | Kutzer and Hinaydy, 1969; Kutzer et al., 1987,1988; Rehbein and Winter, 1999 |  |  |  |
| 14 | *Varestrongylus sagittatus* |  |  |  |  |  |  |  | Kutzer and Hinaidy, 1969; Prosl and Kutzer, 1982 | Rehbein et al., 2014 | Rehbein, 2010 |
| 15 | *Cystocaulus ocreatus* |  | Kutzer, 1969 | Hille, 2004; Kutzer, 1969 |  | Kutzer, 1969 |  |  |  |  |  |
| 16 | *Elaphostrongylus cervi* |  |  |  |  |  |  | Kutzer and Prosl, 1975; | Brugger, 1996; Rehbein, 2010; Prosl and Kutzer, 1982 | Rehbein, 2010 |  |
| 17 | *Muellerius capillaris* |  | Kutzer, 1969 | Hille, 2004; Kutzer and Hinaidy, 1969 | Kutzer and Hinaidy, 1969; Prosl, 1973 | Kutzer, 1969 | Feldbacher, 1979; Kutzer and Hinaidy, 1969 |  |  |  |  |
| 18 | *Muellerius tenuispiculatus* |  |  |  | Kutzer and Hinaidy, 1969 |  | Feldbacher, 1979; Kutzer and Hinaidy, 1969 |  |  |  |  |
| 19 | *Neostrongylus linearis* |  | Kutzer, 1969; Rehbein and Winter, 1999 | Hille, 2004; Kutzer and Hinaidy, 1969 | Kutzer and Hinaidy, 1969; Prosl, 1973 |  | Feldbacher, 1979; Kutzer and Hinaidy, 1969 |  |  |  |  |
| 20 | *Protostrongylus austriacus* |  |  |  | Prosl, 1973 |  | Feldbacher, 1979; Kutzer and Hinaidy, 1969 |  |  |  |  |
| 21 | *Protostrongylus brevispiculum* |  | Kutzer, 1969 |  |  |  |  |  |  |  |  |
| 22 | *Protostrongylus rufescens* |  | Kutzer, 1969; Rehbein and Winter, 1999 | Hille, 2004; Kutzer and Hinaidy, 1969 | Kutzer and Hinaidy, 1969; Prosl, 1973 | Kutzer, 1969 |  |  |  |  |  |
| 23 | *Protostrongylus rupicaprae* |  |  |  | Kutzer and Hinaidy, 1969 |  | Feldbacher, 1979; Kutzer, 1969; Prosl, 1973 |  |  |  |  |
|  | **Dictyocaulidae** |  |  |  |  |  |  |  |  |  |  |
| 24 | *Dictyocaulus capreolus* |  |  |  |  |  |  | Schwarz et al., 2011 |  |  |  |
| 25 | *Dictyocaulus eckerti* |  |  |  |  |  |  | Rehbein, 2010 | Rehbein, 2010 | Rehbein et al., 2014 | Rehbein, 2010; Rehbein and Visser, 2007 |
| 26 | *Dictyocaulus filaria* |  | Kutzer, 1969; Rehbein and Winter, 1999 | Hille, 2004; Kutzer and Hinaidy, 1969 |  | Kutzer, 1969; Prosl, 2009 (pers. comm.) | Kutzer and Hinaidy, 1969 |  |  |  |  |
| 27 | *Dictyocaulus viviparus* | Kutzer, 1988; Böhm, 1987 |  |  |  |  |  | Kutzer and Hinaidy, 1969; Kutzer et al., 1987, 1988; Rehbein and Winter, 1999 | Kutzer and Hinaidy, 1969; Prosl and Kutzer, 1982 | Kutzer and Hinaidy, 1969 | Prosl, 1973 |
|  | **Ancylostomatidae** |  |  |  |  |  |  |  |  |  |  |
| 28 | *Bunostomum phlebotomum* | Böhm, 1987; Kutzer, 1988; Velarde, 1977; Karlinger, 1987 |  |  |  |  | Kutzer, 1988 |  |  |  |  |
| 29 | *Bunostomum trigonocephalum* |  | Brugger, 1996; Kutzer, 1988 | Hille, 2004; Kutzer and Hinaidy, 1969 | Kutzer and Hinaidy, 1969; Prosl, 1973 | Kutzer, 1988 | Kutzer, 1988 | Kutzer and Hinaidy, 1969 | Kutzer, 1988 |  |  |
|  | **Chabertiidae** |  |  |  |  |  |  |  |  |  |  |
| 30 | *Chabertia ovina* | Kutzer, 1969; Prosl, 2009 (pers. comm.) | Brugger, 1996; Kutzer, 1969; Prosl, 2009 (pers. comm.) | Hille, 2004; Kutzer and Hinaidy, 1969 | Kutzer and Hinaidy, 1969; Prosl, 1973; Prosl and Reiter, 1984 | Kutzer, 1969 | Brugger, 1996; Feldbacher, 1979; Kutzer and Hinaidy, 1969; Prosl and Reiter, 1984 | Kutzer and Hinaidy, 1969; Rehbein, 2010; Rehbein and Winter, 1999; Schwarz et al., 2011 | Kutzer and Hinaidy, 1969; Prosl, 2009 (pers. comm.); Rehbein, 2010 | Kutzer and Hinaidy, 1969; Prosl, 2009 (pers. comm.) | Brugger, 1996; Prosl, 1973 |
| 31 | *Oesophagostomum radiatum* | Kutzer, 1988; Prosl, 2009 (pers. comm.); Velarde, 1977 |  |  |  |  | Kutzer, 1988 | Brugger, 1996; Kutzer, 1988; Prosl, 2009 (pers. comm.) | Kutzer, 1969; Prosl, 2009 (pers. comm.) |  | Prosl, 1973 |
| 32 | *Oesophagostomum sikae* |  |  |  |  |  |  | Rehbein, 2010 | Kutzer and Hinaidy, 1969; Brugger, 1996 | Kutzer and Hinaidy, 1969; Rehbein et al., 2014 | Rehbein, 2010; Rehbein and Visser, 2007 |
| 33 | *Oesophagostomum venulosum* | Kutzer, 1988; Prosl, 2009 (pers. comm.); Velarde, 1977 | Brugger, 1996; Kutzer, 1969; Prosl, 2009 (pers. comm.) | Hille, 2004; Kutzer and Hinaidy, 1969 | Brugger, 1996; Kutzer and Hinaidy, 1969; Prosl, 1973; Prosl and Reiter, 1984 | Kutzer, 1969 | Brugger, 1996; Feldbacher, 1979; Kutzer and Hinaidy, 1969; Prosl and Reiter, 1984 | Brugger, 1996; Kutzer and Hinaidy, 1969; Kutzer et al., 1987, 1988; Prosl, 2009 (pers. comm.); Rehbein, 2010; Schwarz et al., 2011 | Brugger, 1996; Kutzer and Hinaidy, 1969 | Kutzer, 1988; Prosl, 2009 (pers. comm.); Rehbein et al., 2014 | Rehbein, 2010; Rehbein and Visser, 2007 |
|  | **Trichostrongylidae** |  |  |  |  |  |  |  |  |  |  |
| 34 | *Cooperia curticei* |  | Kutzer, 1988 |  |  |  |  |  |  |  |  |
| 35 | *Cooperia oncophora* | Kutzer, 1969; Velarde, 1977 | Brugger, 1996; Kutzer, 1988 |  |  |  |  |  | Kutzer, 1969 |  |  |
| 36 | *Cooperia pectinata* |  |  | Hille, 2004; Kutzer and Hinaidy, 1969 |  |  |  | Kutzer and Hinaidy, 1969; Brugger, 1996 | Kutzer and Hinaidy, 1969; Brugger 1996 | Kutzer, 1988; Rehbein et al., 2014 | Rehbein, 2010; Rehbein and Visser, 2007 |
| 37 | *Cooperia punctata* | Kutzer, 1988; Velarde, 1977 |  |  |  |  |  | Kutzer et al., 1987, 1988; Prosl, 1973 |  |  |  |
| 38 | *Haemonchus contortus* | Böhm, 1987; Kutzer, 1969; Prosl, 2009 (pers. comm.); Velarde, 1977 | Brugger, 1996; Kutzer, 1969; Prosl, 2009 (pers. comm.) | Hille, 2004; Kutzer and Hinaidy, 1969; Prosl and Reiter, 1984 | Brugger, 1996; Kutzer and Hinaidy, 1969; Prosl, 1973; Prosl, 2009 (pers. comm.) | Kutzer, 1969 | Feldbacher, 1979; Kutzer and Hinaidy, 1969; Prosl, 1973; Prosl and Reiter, 1984 | Brugger, 1996; Kutzer and Hinaidy, 1969; Kutzer et al., 1987, 1988; Prosl, 2009 (pers. comm.); Rehbein, 2010; Schwarz et al., 2011 | Kutzer and Hinaidy, 1969; Prosl, 2009 (pers. comm.); Rehbein, 2010 | Kutzer and Hinaidy, 1969 | Rehbein, 2010; Rehbein and Visser, 2007 |
| 39 | *Marshallagia marshalli* |  | Brugger, 1996; Kutzer, 1988; Prosl, 2009 (pers. comm.) |  | Brugger, 1996; Kutzer, 1969; Prosl and Reiter, 1984; Prosl, 1973 | Kutzer, 1988; Prosl, 2009 (pers. comm.) | Feldbacher, 1979; Kutzer and Hinaidy, 1969; Prosl, 1973; Prosl and Reiter, 1984 |  |  |  |  |
| 40 | *Ostertagia asymmetrica* |  |  |  |  |  |  | Kutzer and Hinaidy, 1969; Kutzer et al., 1987, 1988 | Kutzer and Hinaidy, 1969 | Kutzer and Hinaidy, 1969; Rehbein et al., 2014 | Rehbein, 2010 |
| 41 | *Ostertagia* *boehmi* | Kutzer, 1988; Prosl, 2009 (pers. comm.); Velarde, 1977 | Brugger, 1996; Kutzer, 1969; Prosl, 2009 (pers. comm.) | Hille, 2004; Kutzer, 1969 | Kutzer, 1988; Prosl and Reiter, 1984 |  | Brugger, 1996; Feldbacher, 1979; Kutzer and Hinaidy, 1969; Prosl, 1973; Prosl, 2009 (pers. comm.); Prosl and Reiter, 1984 | Böhm, 1987; Brugger, 1996; Kutzer and Hinaidy, 1969; Kutzer et al., 1987, 1988; Prosl, 2009 (pers. comm.); Rehbein, 2010; Schwarz et al., 2011 | Kutzer and Hinaidy, 1969; Prosl, 2009 (pers. comm.); Rehbein, 2010 | Kutzer, 1988; Rehbein et al., 2014 | Rehbein, 2010; Rehbein and Visser, 2007 |
| 42 | *Ostertagia circumcincta* | Kutzer, 1988; Prosl, 2009 (pers. comm.) | Brugger, 1996; Kutzer, 1969; Prosl, 2009 (pers. comm.) | Hille, 2004; Kutzer and Hinaidy, 1969 | Kutzer and Hinaidy, 1969; Prosl, 1973; Prosl and Reiter, 1984; Prosl, 2009 (pers. comm.) | Kutzer, 1969; Prosl, 2009 (pers. comm.) | Feldbacher, 1979; Kutzer and Hinaidy, 1969; Prosl, 1973; Prosl, 2009 (pers. comm.); Prosl and Reiter, 1984 | Brugger, 1996; Kutzer and Hinaidy, 1969; Kutzer et al., 1987, 1988; Prosl, 2009 (pers. comm.) | Kutzer, 1969 |  |  |
| 43 | *Ostertagia dahurica* |  | Kutzer, 1969 |  |  |  |  |  |  |  |  |
| 44 | *Ostertagia drozdzi* |  |  |  |  |  |  |  |  | Kutzer, 1988; Rehbein et al., 2014 |  |
| 45 | *Ostertagia houdemeri* |  |  |  |  |  |  | Rehbein, 2010 | Rehbein, 2010 |  | Rehbein, 2010 |
| 46 | *Ostertagia leptospicularis* | Kutzer, 1988; Prosl, 2009 (pers. comm.); Velarde, 1977 | Brugger, 1996; Kutzer, 1988; Prosl, 2009 (pers. comm.) | Hille, 2004; Kutzer and Hinaidy, 1969 | Brugger, 1996; Kutzer and Hinaidy, 1969; Prosl, 1973; Prosl and Reiter, 1984; Prosl, 2009 (pers. comm.) |  | Brugger, 1996; Feldbacher, 1979; Kutzer and Hinaidy, 1969; Prosl, 1973; Prosl, 2009 (pers. comm.); Prosl and Reiter, 1984 | Brugger, 1996; Kutzer and Hinaidy, 1969; Kutzer et al., 1987, 1988; Prosl, 2009 (pers. comm.); Rehbein, 2010; Schwarz et al., 2011 | Kutzer and Hinaidy, 1969; Prosl, 2009 (pers. comm.); Rehbein, 2010 | Kutzer, 1969; Rehbein et al., 2014 | Prosl, 1973; Brugger, 1996 |
| 47 | *Ostertagia occidentalis** |  | Kutzer, 1988 |  | Brugger, 1996; Kutzer and Hinaidy, 1969; Prosl, 1973; Prosl and Reiter, 1984 |  | Brugger, 1996; Feldbacher, 1979; Kutzer and Hinaidy, 1969; Prosl, 1973; Prosl and Reiter, 1984 | Kutzer and Hinaidy, 1969 | Kutzer, 1969 |  |  |
| 48 | *Ostertagia ostertagi* | Kutzer, 1969; Prosl, 2009 (pers. comm.); Velarde, 1977 | Kutzer, 1988; Prosl, 2009 (pers. comm.) | Prosl, 1973 | Kutzer and Hinaidy, 1969; Prosl, 1973; Prosl and Reiter, 1984; Prosl, 2009 (pers. comm.) | Kutzer, 1988 | Feldbacher, 1979; Kutzer and Hinaidy, 1969; Prosl, 1973; Prosl, 2009 (pers. comm.); Prosl and Reiter, 1984 | Brugger, 1996; Kutzer and Hinaidy, 1969; Kutzer et al., 1987, 1988; Prosl, 2009 (pers. comm.); Rehbein, 2010 | Kutzer and Hinaidy, 1969; Prosl, 2009 (pers. comm.) |  |  |
| 49 | *Skrjabinema ovis* |  | Kutzer, 1988 | Hille, 2004: Kutzer and Hinaidy, 1969 | Kutzer, 1988; Prosl, 1973 | Kutzer, 1988 | Brugger, 1996 | Kutzer and Hinaidy, 1969 | Kutzer, 1969 |  |  |
| 50 | *Skrjabinema rupicaprae* |  |  |  |  |  | Kutzer and Hinaidy, 1969; Prosl, 1973 |  |  |  |  |
| 51 | *Trichostrongylus askivalli* |  |  |  |  |  | Feldbacher, 1979; Kutzer, 1969 | Brugger, 1996; Kutzer and Hinaidy, 1969 | Brugger, 1996; Kutzer and Hinaidy, 1969; | Kutzer, 1988; Rehbein et al., 2014 | Rehbein, 2010; Rehbein and Visser, 2007 |
| 52 | *Trichostrongylus axei* | Kutzer, 1969; Prosl, 2009 (pers. comm.); Velarde, 1977 | Brugger, 1996; Kutzer, 1969; Prosl, 2009 (pers. comm.) | Hille, 2004; Kutzer and Hinaidy, 1969 | Brugger, 1996; Kutzer and Hinaidy, 1969; Prosl, 1973; Prosl and Reiter, 1984 | Kutzer, 1969 | Brugger, 1996; Feldbacher, 1979; Kutzer and Hinaidy, 1969; Prosl, 1973; Prosl, 2009 (pers. comm.); Prosl and Reiter, 1984 | Brugger, 1996; Kutzer and Hinaidy, 1969; Kutzer et al., 1987, 1988; Prosl, 2009 (pers. comm.); Rehbein, 2010; Schwarz et al., 2011 | Kutzer and Hinaidy, 1969; Prosl, 2009 (pers. comm.); Rehbein, 2010 | Kutzer, 1988 | Rehbein, 2010; Rehbein and Visser, 2007 |
| 53 | *Trichostrongylus capricola* | Prosl, 2009 (pers. comm.) | Brugger, 1996; Kutzer, 1988; Prosl, 2009 (pers. comm.) | Hille, 2004; Kutzer and Hinaidy, 1969 | Brugger, 1996; Kutzer and Hinaidy, 1969; Prosl, 1973; Prosl and Reiter, 1984 | Kutzer, 1969 | Brugger, 1996; Feldbacher, 1979; Kutzer and Hinaidy, 1969; Prosl, 2009 (pers. comm.); Prosl and Reiter, 1984 | Brugger, 1996; Kutzer et al., 1987, 1988; Prosl, 2009 (pers. comm.); Schwarz et al., 2011 | Kutzer, 1969; Prosl, 2009 (pers. comm.) |  |  |
| 54 | *Trichostrongylus colubriformis* | Prosl, 2009 (pers. comm.) | Brugger, 1996; Kutzer, 1969; Prosl, 2009 (pers. comm.) | Hille, 2004; Kutzer and Hinaidy, 1969 | Brugger, 1996; Kutzer and Hinaidy, 1969; Prosl and Reiter, 1984; Prosl, 1973 | Kutzer, 1969 | Brugger, 1996; Feldbacher, 1979; Kutzer, 1988; Prosl, 2009 (pers. comm.); Prosl and Reiter, 1984 | Brugger, 1996; Kutzer et al., 1987, 1988; Kutzer and Hinaidy, 1969; Prosl, 2009 (pers. comm.); Schwarz et al., 2011 | Prosl, 2009 (pers. comm.) | Kutzer, 1988 |  |
| 55 | *Trichostrongylus longispicularis* | Kutzer, 1988 | Brugger, 1996; Kutzer, 1988 |  | Brugger, 1996 |  | Brugger, 1996 | Brugger, 1996; Kutzer, 1988 |  |  |  |
| 56 | *Trichostrongylus probolurus* |  |  |  |  |  |  |  |  | Prosl, 1973 |  |
| 57 | *Trichostrongylus retortaeformis* |  |  |  |  |  |  | Kutzer et al., 1987, 1988 |  |  |  |
| 58 | *Trichostrongylus vitrinus* |  | Brugger, 1996; Kutzer, 1969 | Hille, 2004; Kutzer and Hinaidy, 1969 | Brugger, 1996; Kutzer and Hinaidy, 1969; Prosl, 1973; Prosl and Reiter, 1984 | Kutzer, 1969 | Brugger, 1996; Feldbacher, 1979; Kutzer and Hinaidy, 1969; Prosl and Reiter, 1984 | Brugger, 1996; Kutzer, 1988 |  |  |  |
|  | **Molineidae** |  |  |  |  |  |  |  |  |  |  |
| 59 | *Nematodirus abnormalis* |  | Brugger, 1996 |  | Brugger, 1996; Kutzer, 1988; Prosl and Reiter, 1984 |  | Brugger, 1996; Kutzer, 1969; Prosl and Reiter, 1984 |  |  |  |  |
| 60 | *Nematodirus battus* |  | Kutzer, 1988; Rehbein and Winter, 1999 |  |  |  |  |  |  | Rehbein et al., 2014 |  |
| 61 | *Nematodirus europaeus* |  |  |  |  |  | Brugger, 1996; Feldbacher, 1979; Kutzer, 1969; Prosl and Reiter, 1984 | Brugger, 1996; Kutzer et al., 1987, 1988; Kutzer, 1988; Rehbein, 2014; Schwarz et al., 2011 | Kutzer, 1969 |  |  |
| 62 | *Nematodirus filicollis* | Kutzer, 1969; Prosl, 2009 (pers. comm.) | Brugger, 1996; Kutzer, 1969; Prosl, 2009 (pers. comm.) | Hille, 2004; Kutzer and Hinaidy, 1969 | Brugger, 1996; Kutzer and Hinaidy, 1969; Prosl, 1973; Prosl and Reiter, 1984 | Kutzer, 1969 | Brugger, 1996; Feldbacher, 1979; Kutzer and Hinaidy, 1969; Prosl, 2009 (pers. comm.); Prosl and Reiter, 1984 | Kutzer and Hinaidy, 1969 | Kutzer and Hinaidy, 1969; Prosl, 2009 (pers. comm.) | Kutzer and Hinaidy, 1969; Prosl, 1973 |  |
| 63 | *Nematodirus helvetianus* | Böhm, 1987; Kutzer, 1969; Prosl, 2009 (pers. comm.); Velarde, 1977 | Kutzer, 1988 |  |  |  |  |  |  |  |  |
| 64 | *Nematodirus oiratianus* |  |  |  | Brugger, 1996 |  | Brugger, 1996 |  |  |  |  |
| 65 | *Nematodirus roscidus* | Kutzer, 1988 |  |  |  |  |  | Brugger, 1996; Kutzer, 1988 | Kutzer and Hinaidy, 1969 | Kutzer, 1988; Prosl, 2009 (pers. comm.); Rehbein et al., 2014 | Rehbein, 2010; Rehbein and Visser, 2007 |
| 66 | *Nematodirus rupicaprae* |  |  |  | Brugger, 1996 |  | Brugger, 1996 |  |  |  |  |
| 67 | *Nematodirus spathiger* | Kutzer, 1969 | Brugger, 1996; Kutzer, 1969 | Kutzer, 1969 |  | Kutzer, 1969 | Brugger, 1996; Feldbacher, 1979; Kutzer and Hinaidy, 1969; Prosl and Reiter, 1984 | Kutzer, 1988 |  |  |  |
|  | **Trichuridae** |  |  |  |  |  |  |  |  |  |  |
| 68 | *Capillaria bovis* | Kutzer, 1988 | Kutzer, 1988 | Hille, 2004; Kutzer and Hinaidy, 1969 | Kutzer and Hinaidy, 1969; Prosl, 1973 | Kutzer, 1988 | Kutzer and Hinaidy, 1969 | Brugger, 1996; Kutzer and Hinaidy, 1969 | Brugger, 1996; Kutzer and Hinaidy, 1969 | Kutzer and Hinaidy, 1969; Rehbein et al., 2014 |  |
| 69 | *Trichuris capreoli* | Kutzer, 1988 |  | Hille, 2004; Kutzer and Hinaidy, 1969 |  |  | Buchacher-Tonitz, 1987; Kutzer, 1988 | Brugger, 1996; Kutzer and Hinaidy, 1969; Prosl, 2009 (pers. comm.); Schwarz et al., 2011 | Kutzer, 1988; Prosl, 2009 (pers. comm.) | Kutzer, 1988 |  |
| 70 | *Trichuris discolor* | Böhm, 1987; Gutierres, 1971 ; Kutzer, 1988; Prosl, 2009 (pers. comm.); Velarde, 1977 |  |  |  |  |  |  |  |  |  |
| 71 | *Trichuris globulosa* |  | Kutzer, 1988; Prosl, 2009 (pers. comm.) | Kutzer and Hinaidy, 1969 | Hille, 2004; Kutzer, 1988; Prosl and Reiter, 1984 |  | Feldbacher, 1979; Kutzer and Hinaidy, 1969; Prosl and Reiter, 1984 | Brugger, 1996; Kutzer and Hinaidy, 1969; Prosl, 2009 (pers. comm.); Rehbein, 2010 | Kutzer, 1969; Brugger, 1996 | Kutzer, 1988; Rehbein et al., 2014 | Rehbein, 2010; Rehbein and Visser, 2007 |
| 72 | *Trichuris ovis* |  | Brugger, 1996; Kutzer, 1988; Prosl, 2009 (pers. comm.) | Hille, 2004; Kutzer and Hinaidy, 1969 | Brugger, 1996; Kutzer and Hinaidy, 1969; Prosl, 1973; Prosl and Reiter, 1984 | Kutzer, 1969 | Brugger, 1996; Feldbacher, 1979; Kutzer and Hinaidy, 1969; Prosl and Reiter, 1984 | Brugger, 1996; Kutzer and Hinaidy, 1969; Kutzer et al., 1987, 1988; Rehbein, 2010 | Kutzer, 1988 | Kutzer, 1988 |  |
| 73 | *Trichuris skrjabini* |  | Brugger, 1996; Kutzer, 1988 |  | Kutzer, 1969; Prosl, 1973 |  | Brugger, 1996; Feldbacher, 1979; Kutzer, 1988 | Brugger, 1996; Kutzer and Hinaidy, 1969 |  |  |  |

** Ostertagia occidentalis*: minor monomorph of *Marshallagia marshalli.*

| **References** |
| --- |
| Böhm, N. (1987). Erhebungen zum Endoparasitenbefall der Rinder in der Steiermark im Rahmen des Forschungsprojektes “Rindergesundheitsdienst.” Doctoral Thesis, Vetmeduni Vienna, Austria. |
| Brugger, A. (1996). Vergleichende Untersuchung zur Magen-Darm-Nematodenfauna der Wild-und Hauswiederkäuer in Osttirol. Doctoral Thesis, Vetmeduni Vienna, Austria. |
| Feichtenschlager, C., Hinney, B., Klose, S., Tichy, A., Tix, A., Strobl, L., and Krametter-Frötscher, R. (2014). Vorkommen von Helminthen beim kleinen Wiederkäuer in der Steiermark mit besonderer Berücksichtigung der Wirksamkeit von Benzimidazolen und makrozyklischen Laktonen. Wien Tierärztl Monatsschr. 101(11–12). |
| Feldbacher, P. (1979). Zusammenhänge zwischen einigen Stoffwechselparametern, Endoparasitenbefall und Räude der Gämse. Doctoral Thesis, Vetmeduni Vienna, Forschungsinstitut für Wildtierkunde, Austria. |
| Gutierres, V. T. (1971). Die Gastrointestinalparasiten des Rindes in Österreich. Doctoral Thesis, Vetmeduni Vienna, Austria. |
| Halabi, M., Pernegger, C., Brinninger, G., and Auer, H. (1997). *Fasciola hepatica* in Österreich - ein Fallbericht. Mitt. Österr. Ges. Tropenmed. Parasitol. 19, 137–142. |
| Hille, G. (2004). Untersuchungen zum Endoparasitenbefall des Muffelwildes in Thüringen. Doctoral Thesis, Univ. Leipzig, Faculty Vet. Med., Germany. |
| Hinaidy, H. K. (1983). *Dicrocoelium suppereri* nomen novum (syn. *D. orientalis* Sudarikov et Ryjikov 1951), ein neuer Trematode für die Parasitenfauna Österreichs. Zentralbl. Vetmed. B. 30(1–10), 576–589. |
| Karlinger, B. (1987). Zum Befall von jungen Milchkühen mit Lungen-und Magen-Darmwürmern in alpinen Rinderbeständen. Doctoral Thesis, Vetmeduni Vienna, Austria. |
| Kutzer, E. (1969). Beziehungen und Übertragungsmöglichkeiten zwischen den Parasiten der Haus- und Wildwiederkäuer. Anblick 24, 7–12. |
| Kutzer, E. (1988). Bedeutung Parasitärer Wechselinfektionen bei Haus- und Wildwiederkäuern. Monatsschr. Vetmed. 43, 577–580. |
| Kutzer, E., and Hinaidy, H. K. (1969). Die Parasiten der wildlebenden Wiederkäuer Österreichs. Zeitschr. Prasitenk. 32(4), 354–368. |
| Kutzer, E., and Prosl, H. (1975). Contribution to the knowledge of *Elaphostrongylus cervi* Cameron, 1931. I. Morphology and diagnosis. Wien. Tierärztl. Monatsschr. 62, 258–266. |
| Kutzer, E., Sugár, L., and Buchacher-Tonitz, S., (1987) Beiträge zur Parasitenfauna der wildlebenden Wiederkäuer Ungarns. I. Zur Parasitenfauna von Rehkitzen und Jungrehen (*Capreolus c. capreolus*). Parasitol. Hung. 20, 81-90. |
| Kutzer, E., Sugár, L., and Buchacher-Tonitz, S., (1988) Beiträge zur Parasitenfauna der wildlebenden Wiederkäuer Ungarns. II. Aufbauentwicklung des Parasitenbefalls bei Rehen (*Capreolus c. capreolus*). Parasitol. Hung. 21, 85-97. |
| Otranto, D., Rehbein, S., Weigl, S., Cantacessi, C., Parisi, A., Lia, R.P., et al. (2007). Morphological and molecular differentiation between *Dicrocoelium dendriticum* (Rudolphi, 1819) and *Dicrocoelium chinensis* (Sudarikov and Ryjikov, 1951) Tang and Tang, 1978 (Platyhelminthes: Digenea). Acta Trop. 104(2–3), 91–98. <https://doi.org/10.1016/j.actatropica.2007.07.008> |
| Prosl, 2009 (pers. comm.) |
| Prosl, H. (1973). Beiträge zur Parasitenfauna der wildlebenden Wiederkäuer Österreichs. Doctoral Thesis, Vetmeduni Vienna, Austria. |
| Prosl, H., and Kutzer, E. (1982). Jahresrhythmus in der Larvenausscheidung von *Dictyocaulus viviparus*, *Varestrongylus sagittatus* und *Elaphostrongylus cervi* bei Rotwild (Cervus elaphus). Angew. Parasitol. 23(1), 9-14. |
| Prosl, H., and Reiter, I. (1984). Vergleichende Untersuchungen zur Gastrointestinal-Nematodenfauna von Gemse *(Rupicapra rupicapra*) und Steinbock (*Capra ibex*). Z. Jagdwiss. 30(2), 89–100. |
| Rehbein, S. (2010). Die Endoparasiten des Sikawildes in Deutschland und Österreich. Doctoral Thesis. Paris London University Salzburg. |
| Rehbein, S., and Visser, M. (2007). The endoparasites of Sika deer (*Cervus nippon*) in Austria. Wien. Klin. Wochenschr. 119(Suppl. 3), 96–101. <https://doi.org/10.1007/s00508-007-0865-5> |
| Rehbein, S., Visser, M., and Winter, R. (1999). Ein Beitrag zur Kenntnis des Parasitenbefalls von Bergschafen aus dem Oberpinzgau (Salzburg). Mitt. Österr. Ges. Tropenmed. Parasitol. 21, 99–106. |
| Rehbein, S., Visser, M., Jekel, I., and Silaghi, C. (2014). Endoparasites of the fallow deer (*Dama dama*) of the Antheringer Au in Salzburg, Austria. Wien. Klin. Wochenschr. 126(Suppl. 1), 37–41. <https://doi.org/10.1007/s00508-014-0506-8> |
| Sattmann, H., Hörweg, C., Gaub, L., Feix, A. S., Haider, M., Walochnik, J. (2014). Wherefrom and whereabouts of an alien: The American liver fluke *Fascioloides magna* in Austria: An overview. Wien. Klin. Wochenschr. 126 (1), 23–31. <https://doi.org/10.1007/s00508-014-0499-3> |
| Schwarz, L., Frena, M., Skalicky, M., and Prosl, H. (2011). Endoparasitenbefall von Rehen in einem Revier in Niederösterreich. Wien. Tierärztl. Monatsschr. 98(11–12), 285–291. |
| Velarde, O. (1977). Weitere Untersuchungen zur Erfassung der Helminthen von Labmagen und Darm des Rindes in Österreich. Doctoral Thesis, Vetmeduni Vienna, Austria. |
